# Augmenting mindfulness training through neurofeedback: a pilot study of the pre-post changes on resting-state functional connectivity in typically developing adolescents

**DOI:** 10.3389/fnins.2024.1397234

**Published:** 2024-10-30

**Authors:** Kelly T. Cosgrove, Aki Tsuchiyagaito, Zsofia P. Cohen, Gabe Cochran, Xiaoqian Yu, Masaya Misaki, Robin L. Aupperle, Manpreet K. Singh, Martin P. Paulus, Namik Kirlic

**Affiliations:** ^1^Laureate Institute for Brain Research, Tulsa, OK, United States; ^2^Department of Psychiatry, University of Colorado School of Medicine, Aurora, CO, United States; ^3^Department of Psychology, Oklahoma State University, Stillwater, OK, United States; ^4^School of Psychology, Wenzhou-Kean University, Zhejiang, China; ^5^School of Community Medicine, University of Tulsa, Tulsa, OK, United States; ^6^Department of Psychiatry and Behavioral Sciences, University of California, Davis, CA, United States

**Keywords:** mindfulness, fMRI neurofeedback, posterior cingulate cortex (PCC), resting-state fMRI, functional connectivity, adolescence

## Abstract

**Background:**

Mindfulness training has been shown to promote positive mental health outcomes and related changes in neural networks such as the default mode network, which has a central node in the posterior cingulate cortex (PCC). Previous work from our group reported on the impact of a novel, neurofeedback augmented mindfulness training (NAMT) task on regulation of PCC hemodynamic activity in typically developing adolescents. The present pilot study aimed to expand on this finding by examining the pre-post changes of the NAMT task on resting-state functional connectivity of the PCC.

**Methods:**

Thirty-one typically developing adolescents (14.77 ± 1.23 years; 45% female) underwent a resting-state functional magnetic resonance imaging scan both before and after completing the NAMT task. A linear mixed effects model was used to assess for changes in functional connectivity of the PCC across the two resting-state runs.

**Results:**

Data did not support the hypothesized decrease in connectivity between the PCC seed and other DMN regions from pre- to post-NAMT task. However, we observed a significant increase in functional connectivity between the PCC and a cluster encompassing the left hippocampus and amygdala following completion of the NAMT task (run 1 Fisher’s *Z* = 0.16; run 2 Fisher’s *Z* = 0.26).

**Conclusion:**

Although preliminary, this finding suggests NAMT has the potential to strengthen connectivity between default mode and salience regions. We speculate that such changed connectivity may facilitate enhanced self-referential and emotional processing in adolescents.

**Clinical trial registration:**

https://clinicaltrials.gov/, identifier NCT04053582.

## 1 Introduction

Mindfulness training, rooted in Buddhist traditions, aims to aid individuals in observing the present moment with openness, acceptance, and non-judgment (Davis and Hayes, 2011; Kabat-Zinn, 2015). Previous research indicates mindfulness training (i.e., attending to experience on purpose and non-judgmentally) can result in several positive outcomes that are protective against mental health symptoms, such as enhanced emotion regulation and attentional control, as well as decreased reactivity (i.e., a reduction in the intensity and frequency of emotionally, physiological, and behavioral responses to stimuli), mental distress and cravings (Basso et al., 2019; Brandtner et al., 2022; Keng et al., 2011). Especially important for adolescents, who may be more susceptible to stress and emotional challenges during this critical developmental period (De Girolamo et al., 2012), mindfulness training may serve as a beneficial preventive strategy. Indeed, evidence suggests that mindfulness training can significantly reduce self-reported symptoms of anxiety and depressed mood in adolescents without diagnosed mental health disorders (Tan and Martin, 2016; Zhou et al., 2020). This population may further be well-suited for mindfulness training due to the neuroplasticity observed in adolescence and associated enhanced learning that can occur during this developmental period (Casey et al., 2011). However, the optimization of mindfulness training for adolescents is hindered by a lack of understanding of its underlying psychological and neurobiological mechanisms of action.

Neuroimaging studies have provided initial insights into the neurocircuitry that may support mindfulness. Findings from both structural and functional imaging studies suggest the key regions associated with mindfulness are those involved in the default mode network [DMN; e.g., medial prefrontal cortex (mPFC), posterior cingulate cortex (PCC), precuneus] and salience network (SN; e.g., insula and amygdala; Hatchard et al., 2017; Marchand, 2014; Sezer et al., 2022). The DMN is thought to support self-relevant processing and social cognition (e.g., theory of mind; Buckner et al., 2008), and the SN is involved in the detection of relevant stimuli in one’s environment that are both external and internal, such as emotions (Menon and Uddin, 2010). Task-based functional magnetic resonance imaging (fMRI) studies of adults who practice mindfulness reveal a pattern of decreased activation within key DMN regions, particularly the PCC, regardless of level of experience with mindfulness and meditation (e.g., Farb et al., 2007; Garrison et al., 2015; Grant et al., 2011). Researchers employing resting-state fMRI (rs-fMRI) have found that mindfulness may alter the functional connectivity (i.e., co-activation of two or more regions) within and between regions of the DMN and SN. Specifically, research has shown decreased amygdala-subgenual ACC resting state functional connectivity (Taren et al., 2015), as well as greater coupling between the PCC, dACC, and dlPFC (Brewer et al., 2011) in adults as a function of mindfulness training. Functional coupling between the insula and the mPFC has been observed in adult novice meditators, while those who completed an 8-week mindfulness course, evidence uncoupling between these regions (Farb et al., 2007). Similarly, greater trait mindfulness was found to be associated with negative correlations between the precuneus/PCC and the insula among adults (Bilevicius et al., 2018). Though nuanced, these rs-fMRI findings highlight a consistent relationship between mindfulness and neural networks involved in attention, emotion processing, and self-awareness. Further research is needed to clarify the relationship between mindfulness training and functional connectivity of the PCC (and the DMN and SN more broadly), particularly in adolescent populations.

To address this, we developed the Neurofeedback Augmented Mindfulness Training (NAMT) task, which combines mindfulness training (secular practice focused on cultivating non-judgmental awareness and presence in the moment) with real-time fMRI neurofeedback to optimize learning and performance, that is, engage with and apply mindfulness practice effectively, as well as improve attentional control and emotional regulation. During the task, participants are guided to focus on their breath while receiving visual feedback of current hemodynamic activation in the PCC, which was selected due to its promise as a neural target of mindfulness training (Brewer and Garrison, 2014). Using the visual feedback, participants are instructed to decrease their PCC activation while completing the breathing exercise. Preliminary work from our group suggests the NAMT task is feasible and tolerable, as evidenced by a strong study completion rate, no associated adverse events, and positive participant ratings of the task (Kirlic et al., 2022). Participants demonstrated decreased activity in the PCC seed as well as other DMN and SN regions during mindfulness training while receiving neurofeedback (compared to a control condition of the NAMT task that involves describing oneself; Kirlic et al., 2022). Another study examined activation in the insular cortex during the NAMT task using an overlapping sample and found that participants exhibited increased activity in the anterior insula and decreased activity in the posterior insula during neurofeedback relative to the control condition of the task (Yu et al., 2022). Similarly, in a recent study by another group of investigators, mindfulness training with fMRI neurofeedback was examined in a sample of nine adolescents with a lifetime history of depression and/or anxiety diagnoses (Zhang et al., 2023). The results of this experiment indicated that mindfulness training was associated with reduced connectivity in DMN, specifically between the seed region sgACC and PCC, and mPFC, as well as increased levels of self-reported state mindfulness from pre- to post-neurofeedback.

In the present pilot study, we aimed to explore the pre-post changes in functional connectivity during rs-fMRI as a function of the NAMT task in a typically developing adolescent sample without psychiatric disorders. Specifically, we were interested in extending on our previous work by examining differences in neurocircuitry after participants complete the NAMT task, relative to before the task. Based on the task’s design, which selectively targets specific neural regions associated with mindfulness practice, we hypothesized that participants would demonstrate an overall decrease in connectivity between the PCC seed and other DMN regions from pre- to post-NAMT task. We also hypothesized the NAMT task would increase connectivity between the PCC seed and regions of the SN, in accordance with existing literature. It is important to note that this pilot study did not include a control group/sham condition, and thus the findings presented within are considered exploratory given the novelty of the NAMT approach.

## 2 Methods

### 2.1 Participants

Participants were derived from the same sample as described in previous reports of the NAMT task (Kirlic et al., 2022; Yu et al., 2022) and included typically developing adolescents between the ages of 13 and 17 years who were enrolled in a larger neuroimaging study of adolescents at the Laureate Institute for Brain Research in Tulsa, OK. Though the larger study also included participants with exposure to early life adversity, here we focus only on healthy adolescents without history of psychiatric disorders. By concentrating on these adolescents, we intended to establish a baseline understanding of how the NAMT task relates to neural connectivity in a normative developmental context. These participants were recruited from the community via flyers and advertisements on the radio, social media, billboards, and a message board through the local school system. Exclusion criteria for the present study included (*i*) history of a mental health, neurological, or developmental disorder; (*ii*) history of traumatic brain injury; (*iii*) current use of psychotropic medications or those that may influence brain function or blood flow (e.g., prophylactic medications for migraines); and (*iv*) any MRI contraindications (e.g., dental braces). Parents/legal guardians provided written informed consent for their child’s participation, and adolescents provided written assent. The study protocol was approved by the Western Institutional Review Board and conducted in accordance with the Declaration of Helsinki. Forty adolescents were enrolled in the study and 36 of these participants successfully completed the fMRI scanning protocol, which for the present analysis included two rs-fMRI runs (one before and one following the NAMT task). Two participants withdrew following missed appointments, and the other two could not be included in the current study due to missing data because of technical difficulties during scanning. Five of the participants with complete scan data were excluded prior to analyses for demonstrating excessive motion [i.e., an average Euclidean norm of six motion parameters (ENORM) ≥ 0.25 (*n* = 3) and/or more than one third of frames censored (*n* = 2)] during either of the two rs-fMRI runs. Thus, the final sample for the present study included 31 adolescents (14.71 ± 1.27 years; 45% female). We note that the sample size for the present study was determined by the overarching trial protocol (for additional information regarding sample characteristics and descriptive statistics of the self-report measures, see Table 1).

**Table 1:** Sample characteristics (*N* = 31).

Variable	*n* (%)
Biological sex	
Female	14 (45)
Male	17 (55)
Race	
White	23 (74)
African American or Black	1 (3)
Native American	2 (6)
Asian	2 (6)
Biracial	3 (10)
Ethnicity	
Hispanic	4 (13)
Non-Hispanic	27 (87)

	*M* (SD)
Age in years	14.77 (1.23)
Body mass index (BMI; kg/m^2^)	22.92 (4.59)

	rs-fMRI Run 1 *M* (SD)	rs-fMRI Run 2 *M* (SD)	*t* (30)	*p*
Average ENORM value	0.06 (0.03)	0.08 (0.04)	1.58	0.12
Peak respiration frequency	0.28 (0.07)	0.27 (0.08)	0.73	0.47

	Pre-scan *M* (SD)	Post-scan *M* (SD)	*t* (30)	*p*
PSS total score	12.29 (4.78)	12.77 (4.93)	1.24	0.22
PANAS-C positive affect score	43.48 (7.48)	42.35 (8.61)	−1.62	0.12
PANAS-C negative affect score	22.39 (5.70)	21.81 (5.34)	−1.19	0.24
SMS mind subscale score	52.29 (10.33)	53.71 (9.70)	1.38	0.18
SMS body subscale score	20.03 (3.99)	21.58 (4.04)	2.70	0.01

The respiration signal, recorded at a 40 Hz sampling frequency, was first bandpass filtered between 0.05 and 2 Hz to remove noise. The power spectrum of the filtered signal was then calculated and smoothed using a Gaussian filter (sigma = 0.011 Hz), after which the peak frequency was identified. ENORM, Euclidean norm of six motion parameters; PSS, Perceived Stress Scale (possible scores range from 0 to 40 with higher scores indicating greater stress); PANAS-C, Positive and Negative Affective Schedule for Children [possible scores range from 12 to 60 (Positive Affect) and 15–75 (Negative Affect), with higher scores indicating stronger affect]; SMS, State Mindfulness Scale [possible scores range from 15 to 75 (Mind) and 6–30 (Body) with higher scores indicating greater state mindfulness].

### 2.2 Study procedures

As noted above, data for the present study were derived from a larger longitudinal study involving one MRI session, and the primary outcome of interest for this investigation was change in resting-state functional connectivity of the PCC from pre- to post-completion of the NAMT task. The study protocol is registered at the U.S. National Institutes of Health on clinicaltrials.gov (identifier #NCT04053582). Data used in the current report were collected at a baseline screening visit (conducted in-person and virtually due to the COVID-19 pandemic) and an MRI visit. At baseline, participants and their caretakers provided information regarding demographics, medical and psychiatric history, pubertal status, and MRI eligibility. At the MRI session, participants completed self-report measures immediately before and after scanning. These included measures of mental health states often targeted by mindfulness interventions (i.e., the Perceived Stress Scale, PSS; a measure of perceived present stress levels with higher scores indicating greater stress; Cohen et al., 1983) and the Positive and Negative Affective Schedule for Children (PANAS-C; a measure of positive and negative state affect with higher scores indicating stronger affect; Hughes and Kendall, 2009) as well as the State Mindfulness Scale (SMS), a measure of perceived attention to and awareness of mental (Mind subscale) and physical (Body subscale) experience with higher scores indicating greater state mindfulness (Tanay and Bernstein, 2013). Each of these measures has been shown to have sufficient internal consistency reliabilities (Cohen et al., 1983; Hughes and Kendall, 2009; Tanay and Bernstein, 2013; Yıldız, 2024). In the present study, the measures demonstrated acceptable test–retest reliabilities from pre- to post-scanning (PSS *r* = 0.90; PANAS-C Positive Affect *r* = 0.89; PANAS-C Negative Affect *r* = 0.88; SMS Mind *r* = 0.84; SMS Body *r* = 0.68).

#### 2.2.1 Neurofeedback augmented mindfulness training task

Before the scan session, adolescents first received a brief psychoeducational introduction into mindfulness, followed by guided mindfulness practice focused on the breath (Brewer et al., 2011; Garrison et al., 2013). Next, adolescents completed the MRI session, which included 8 runs, including an anatomical scan, Resting State scan 1 (Rest-1), Observe (OBS), three Neurofeedback runs (NF-1, NF-2, NF-3), Transfer run (TRS), and Resting State scan 2 (Rest-2). During Rest-1 and Rest-2 (6 min each), participants were instructed to clear their mind and not think about anything while fixating upon a fixation cross. OBS, NF-1, NF-2, NF-3, and TRS runs each lasted 6 min and 56 s. Runs started with a 66-s rest block, followed by alternating Describe (Active Control condition without neurofeedback; 20 s), Focus-on-Breath (MT condition with PCC neurofeedback; 70 s), and Rest (Baseline condition; 30 s) blocks. OBS and TRS runs did not involve neurofeedback (no bar displayed) during the Focus-on-Breath condition.

During the Focus-on-Breath condition (Garrison et al., 2013), adolescents were told to pay attention to the physical sensations of their breath, not trying to change their breathing in any way, and if their attention were to wander to something else, to gently bring their attention back to their breath (Brewer et al., 2011). In the Describe condition, adolescents were shown various adjectives, which they had to mentally categorize as descriptive or not descriptive of them for the entire duration the word was displayed on the screen (Kelley et al., 2002). During the Rest condition, adolescents were shown the cue “Rest” and asked to relax while looking at the display screen.

During neurofeedback runs, adolescents were told that they would see a bar displayed on the screen, representing in real time the relative brain activity in a particular brain region. They were told that the bar may change with the experience of focusing on the breath (i.e., the bar may go blue if they are fully concentrating on their breath, and red if their mind wanders elsewhere). The green bar indicated the target to attain, and adolescents’ goal was to try and see how much they could make the bar change to blue to match the green bar. A more detailed description of the NAMT task procedures and results from this cohort have been reported in a prior publication focused on the success of the task in leading to down regulation of the PCC (Kirlic et al., 2022; cred-NF checklist). While the study design and protocol are consistent between the previous and current publications, it should be noted that the outcome measure for the present manuscript is resting-state functional connectivity data collected before and after neurofeedback rather than hemodynamic activation during the NAMT task.

### 2.3 Rs-fMRI data acquisition and processing

Magnetic resonance imaging was performed using a Discovery MR750 3 Tesla MRI scanner (GE Healthcare, Milwaukee, Wisconsin) with an 8-channel receive-only head coil. Structural MRI scans involved a T1-weighted Magnetization Prepared Rapid Gradient Echo (MPRAGE) imaging sequence with sensitivity encoding (SENSE; acceleration factor: R = 2, scan time = 4 min 59 s; image matrix size: 256 × 256, FOV/slice thickness = 240/1.2 mm, TR/TE = 5.0/1.9 ms, inversion and delay times: TI/TD = 725/1,400 ms, flip angle: 8°, sampling bandwidth: 31.2 kHz, 124 axial slices per volume). The rs-fMRI scans were acquired using single-shot gradient echo-planar imaging (EPI) sequences with SENSE (acceleration R = 2, scan time = 6 min, image matrix size: 128 × 128, FOV/slice thickness = 240/2.9 mm, TR/TE = 2,000/25 ms, acquisition matrix: 96 × 96, flip angle = 90°, 40 axial slices, voxel volume: 1.9 × 1.9 × 2.9 mm^3^). During these scans, participants were instructed to look at a fixation cross and to not think about anything in particular. Lights in the scanner room were off.

The imaging data were preprocessed and analyzed using Analysis of Functional NeuroImages (AFNI) software package (Cox, 1996). The initial three volumes of functional images were discarded. The preprocessing procedures included despiking, physiological noise corrections using RETROICOR (Glover et al., 2000) and respiratory volume per time (Birn et al., 2008), slice timing corrections, motion alignment, as well as nonlinear warping to the MNI template brain with a resampling to a voxel volume of 2 mm^3^ using ANTs^1^ (Avants et al., 2008). Notably, variables used to assess for motion did not differ significantly between the two rs-fMRI runs (Average ENORM values paired *t* = 1.58, *p* = 0.12; Censor fraction paired *t* = 0.97, *p* = 0.34). Peak respiration frequency was also not significantly different between runs (paired *t* = 0.73, *p* = 0.47). Global signal regression (GSR) was not performed on the rs-fMRI data. While GSR is a commonly used technique for removing widespread signals common across the brain, we intentionally chose not to apply it in our analysis as it may remove neuronal signals of interest and introduce artificial anti-correlations (Chen et al., 2012; Murphy and Fox, 2017). We believe our approach adequately corrects for various sources of noise while preserving signals of interest. Spatial smoothing was performed using a Gaussian kernel with a full-width half-maximum (FWHM) of 6 mm within the brain mask, in conjunction with the scaling of the signal to the mean percentage change in each voxel. A general linear model (GLM) analysis was subsequently conducted, with regressors of Legendre polynomial models for slow signal fluctuations, 12 motion parameters (3 shifts, 3 rotations, and their temporal derivatives), censored volumes with ENORM exceeding 0.2 mm, three principal component signals from the ventricles, and average white matter signal (ANATICOR; Jo et al., 2010). The residuals derived from the GLM analysis were employed as the processed fMRI data to compute functional connectivity. Individual-level connectivity analyses were conducted via AFNI by first extracting the timeseries of left PCC seed region (MNI: −5, −55, 23; 7 mm sphere) using 3dMaskAve and then running Fisher’s Z correlations with all other timeseries in the brain using 3dTcorr1D. The mask of the PCC was the same as that used for neurofeedback during the NAMT task (Kirlic et al., 2022), selected based on a meta-analysis investigating functional neuroimaging studies of the DMN (Wang et al., 2018), mindfulness meditation studies, including neurofeedback (Brewer et al., 2011; Garrison et al., 2013), and our pilot testing conducted on an independent sample. This resulted in individual correlation maps for each rs-fMRI run that could then be used for group-level analyses.

### 2.4 Group-level analyses

A linear mixed effects model (LME) was used to assess for changes in functional connectivity with the PCC seed during rs-fMRI from pre- to post-NAMT task. This was performed using the AFNI program 3dLME, with time (i.e., rs-fMRI run 1 or run 2) as the independent variable and PCC connectivity as the dependent variable. Age, sex, and average ENORM values were included as covariates, as was a subject random effect on intercept. This analysis was run across all voxels in the brain, excluding those in the mask of the PCC seed region. No seed-to-seed analyses were performed, as the primary aim of this study was to investigate changes in whole-brain connectivity patterns, rather than restricting the analysis to predefined seed regions. This approach allows for the exploration of broader connectivity changes across multiple brain networks, which may provide a more comprehensive understanding of the effects of mindfulness training. The AFNI program 3dClustSim with the spatial autocorrelation function (acf) option was used to control for multiple comparisons and identify appropriate cluster thresholds (Cox et al., 2017). At a voxel-wise threshold of *p* < 0.001 and with cluster-size correction at *α* = 0.05, the threshold was determined to be 576 mm^3^ (72 voxels). (Team, 2020) was used to calculate descriptive statistics and conduct exploratory correlational analyses to investigate associations between changes in functional connectivity (average Fisher’s *Z*-values extracted for the cluster in the hippocampus/amygdala at each rs-fMRI run and used to calculate a change score) and self-report questionnaires. Effect sizes for the present study are reported to contextualize the results. It should be noted, however, that effect sizes are often inflated in fMRI studies (Yarkoni, 2009) and thus should be considered exploratory and interpreted with caution. No hypotheses were pre-registered for this work.

## 3 Results

### 3.1 Changes in functional connectivity of the PCC

**Figure 1:**
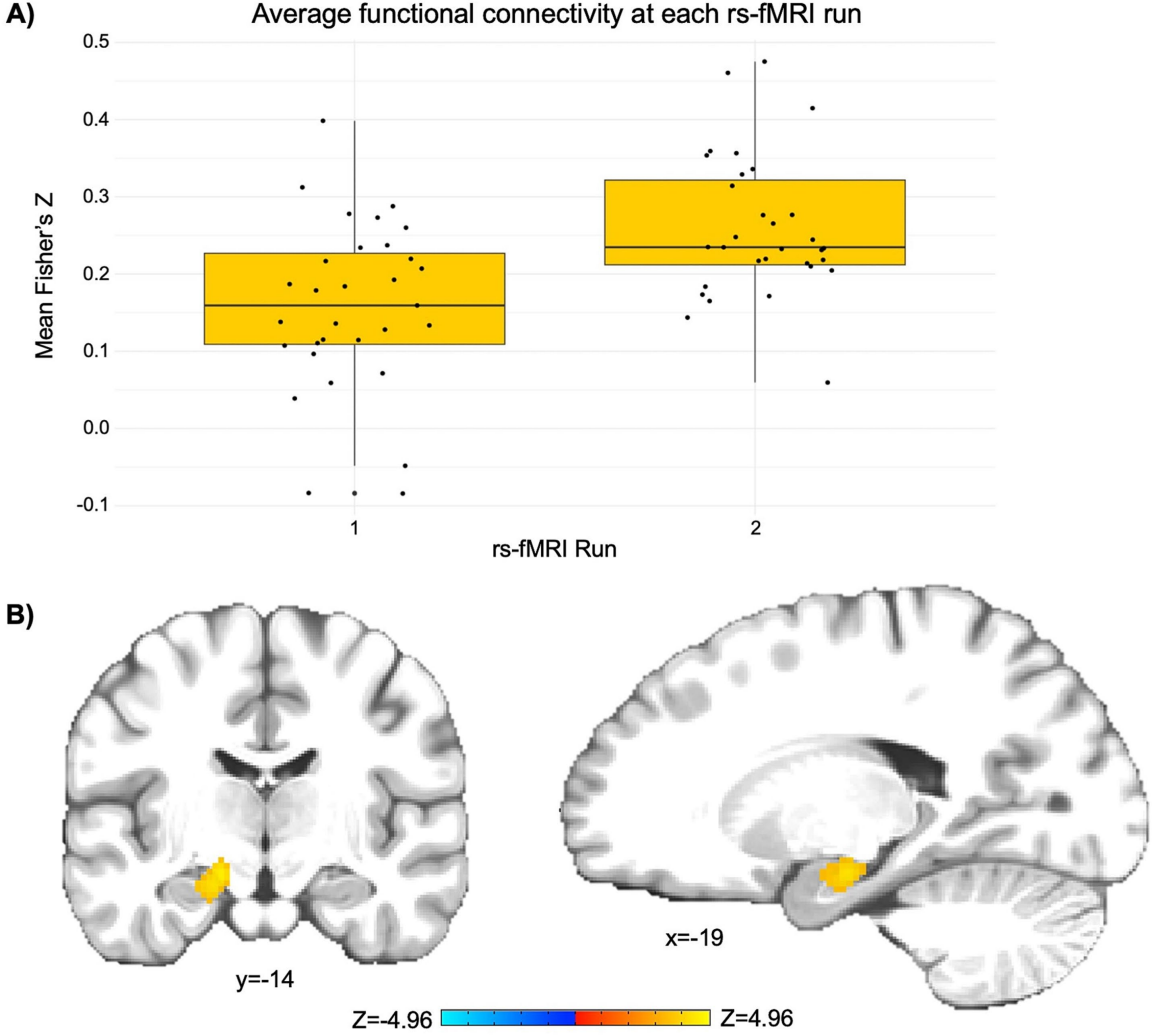
The NAMT task significantly increases functional connectivity between the posterior cingulate cortex (PCC) and left hippocampus/amygdala in adolescents during rest. (A) A depiction of the change in average functional connectivity in the significant cluster (as represented by Fisher’s Z) from pre- to post-NAMT task. These values are reported to aid in the interpretation of results, although it should be noted that they may be overestimates of the true effect size. (B) A visual depiction of the cluster encompassing regions of the left hippocampus and amygdala that demonstrates a significant intervention-related change in connectivity with the PCC seed. Left is left.

The LME revealed a significant cluster (*p* < 0.001) comprised largely of the left rostral hippocampus and regions of the left caudal hippocampus and left medial amygdala (peak *Z* = 4.97; peak MNI coordinates = −23, −17, −17; cluster size = 1,080 mm^3^). This finding indicates a significant increase in functional connectivity between the PCC seed and the left hippocampus and amygdala from pre- to post-NAMT task (rs-fMRI run 1 Fisher’s *Z* = 0.16; rs-fMRI run 2 Fisher’s *Z* = 0.26; see Figure 1).

**Figure 2:**
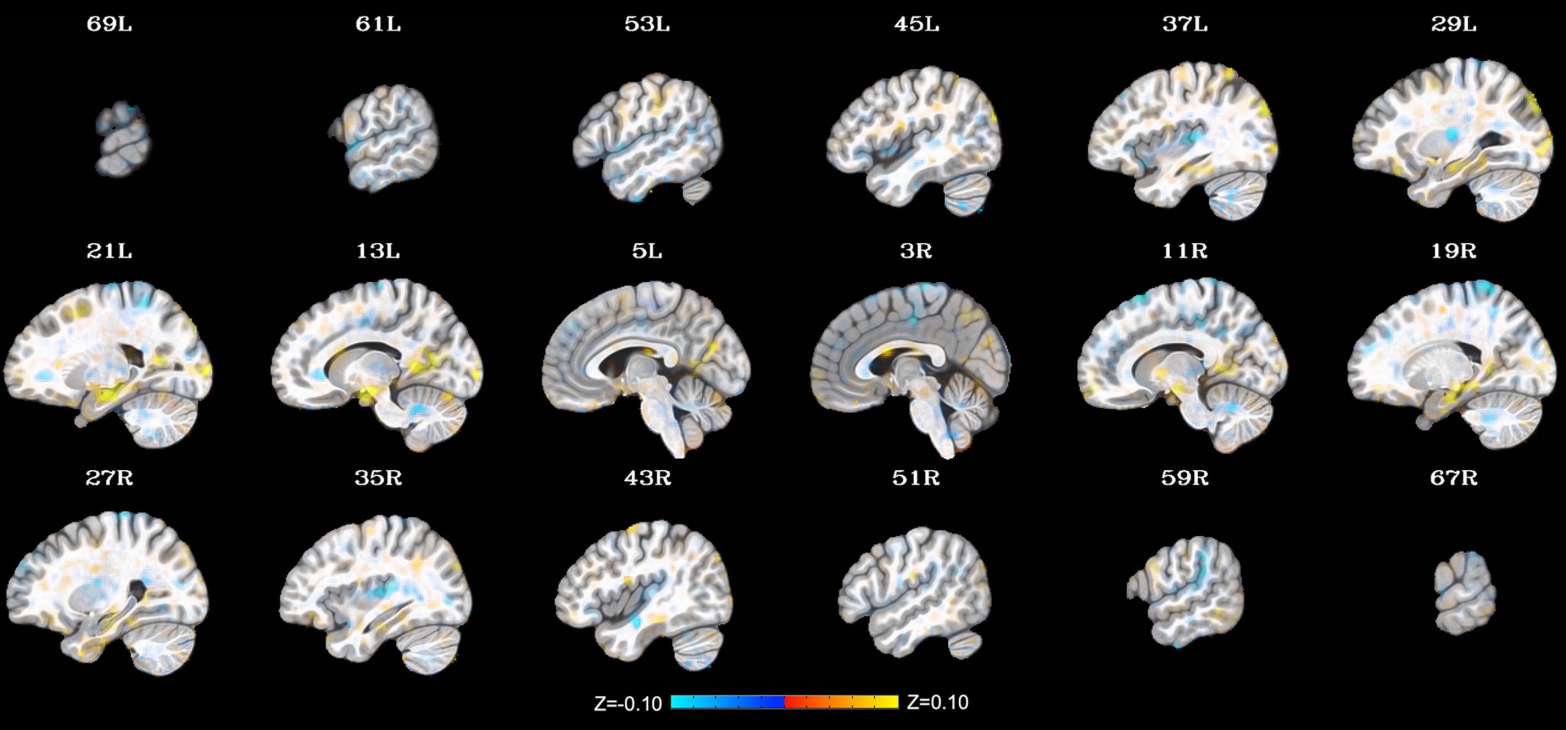
Change in whole-brain functional connectivity with the posterior cingulate cortex seed from pre- to post-NAMT task using a voxel-wise threshold of *p* < 0.001 without cluster-size correction. These results are provided for transparency and reproducibility. L = left, R = right.

Additional clusters were identified through the LME, including relatively small clusters in the left precuneus (peak *Z* = 4.37; peak coordinates = −13, −47, 7; cluster size = 288 mm^3^), left middle occipital gyrus (1. peak *Z* = 4.71; peak coordinates = −17, −101, 1; cluster size = 240 mm^3^ and 2. peak *Z* = 3.81; peak coordinates = −35, −83, 33; cluster size = 224 mm^3^), and left insula/putamen (peak *Z* = −4.60; peak coordinates = −27, −17, 11; cluster size = 240 mm^3^). Notably, these extended findings did not survive cluster-size correction (*p* < 0.001) and thus will not be interpreted herein. They are included to provide additional context to the present findings and generate hypotheses to be tested in future work on this topic. See Figure 2 for a depiction of the whole-brain voxel-wise results without cluster-size correction.

### 3.2 Correlations between changes in connectivity and self-report data

Exploratory correlations were run to assess for associations between the change in PCC-hippocampus/amygdala connectivity and change in self-report measures from pre- to post-scan. None of these relationships were statistically significant (see Table 2). In addition, average ENORM values were not significantly related to PCC-hippocampus/amygdala connectivity in either rs-fMRI run (run 1 *r* = −0.12; run 2 *r* = 0.13).

**Table 2:** Correlations between the change in PCC-hippocampus/amygdala connectivity (rs-fMRI run 1 to run 2) and change in self-report variables (pre- to post-scan; *N* = 31).

	PCC-hippocampus/amygdala connectivity	PSS total score	PANAS-C positive affect score	PANAS-C negative affect score	SMS mind subscale score
PSS total score	−0.28 (*q =* 0.52)				
PANAS-C positive affect score	0.23 (*q =* 0.52)	−0.30 (*q =* 0.52)			
PANAS-C negative affect score	−0.22 (*q =* 0.52)	0.04 (*q =* 0.90)	0.26 (*q =* 0.52)		
SMS mind subscale score	−0.10 (*q =* 0.73)	−0.18 (*q =* 0.53)	0.20 (*q =* 0.52)	0.09 (*q =* 0.73)	
SMS body subscale score	0.17 (*q =* 0.53)	−0.25 (*q =* 0.52)	0.09 (*q =* 0.73)	0.02 (*q =* 0.94)	0.57 (*q =* 0.01)

PSS, Perceived Stress Scale; PANAS-C, Positive and Negative Affective Schedule for Children; SMS, State Mindfulness Scale. q, FDR-adjusted *p*-value.

## 4 Discussion

The overall goal of this pilot study was to examine the pre-post changes in resting-state functional connectivity as a function of a novel, neurofeedback augmented mindfulness training (NAMT) task in a sample of adolescents without diagnosed mental health disorders and move toward identifying the neural correlates associated with mindfulness training. Based on prior literature, we hypothesized that our NAMT task would result in decreased functional connectivity between the PCC and other regions of the DMN from pre- to post-task completion. We also suspected that the task would elicit greater coordination and thus connectivity between the PCC and SN regions. While we found evidence for the second, the first hypothesis was not supported by the data.

This study is one of the first to investigate how mindfulness training with neurofeedback impacts functional connectivity in adolescents and therefore provides novel insights into the neurocircuitry that may support mindfulness. Results revealed increased connectivity between the seed region in the PCC and a cluster encompassing the left hippocampus and amygdala following completion of the NAMT task. Each of these implicated regions is involved in the processing of emotions and memories. As a key node in the DMN, the PCC is active during self-referential thinking. The hippocampus is also linked to the DMN and involved in memory formation and retrieval, particularly autobiographical memory (Burgess et al., 2002). Meanwhile, the amygdala, a subcortical limbic region within the SN, plays a crucial role in emotional processing (Pourtois et al., 2013; Uddin, 2016). While speculative, it is possible that the observed increased connectivity between these regions following the NAMT task might reflect enhanced self-awareness and emotional processing, as participants become more attuned to their internal experiences and emotions. Furthermore, greater connectivity between regions of the DMN and SN might reflect better integration of emotional and cognitive processes, which could enhance the ability to respond adaptively to emotional stimuli, a key aspect of resilience to psychopathology (Weissman et al., 2019).

These findings add to the existing literature regarding the effects of mindfulness training without neurofeedback on DMN and SN regions. For instance, Bremer et al. (2022) assessed resting-state functional connectivity changes in adults who completed either a 31-day mindfulness intervention or an active control condition. The authors found that the mindfulness intervention significantly increased connectivity between the DMN and SN both within subjects (i.e., pre- to post-intervention) and relative to participants in the control condition. Relatedly, a recent meta-analysis of 12 studies of neural changes associated with mindfulness training found evidence for enhanced connectivity between the DMN hub, the PCC, and the middle cingulate located within the SN (Rahrig et al., 2022). Interestingly, our findings are somewhat inconsistent with those of Zhang et al. (2023), who found mindfulness training led to decreased within-DMN connectivity in adolescents with a history of mood or anxiety disorders. The authors interpret their finding to mean that mindfulness training with neurofeedback of the DMN may reduce ruminative thinking in this population. However, there are some notable distinctions between our studies, such as the type of participants [clinical (a lifetime history of MDD and/or anxiety disorders) versus healthy adolescents], sample sizes (*N* = 9 versus *N* = 31), and the specific targets for neurofeedback (sgACC versus PCC activity). Additional studies are therefore needed to clarify the impact of mindfulness training with neurofeedback on resting-state connectivity in adolescents.

We acknowledge that our findings revealed no significant correlations between changes in PCC-hippocampus/amygdala connectivity and changes in self-report measures. These null results are not consistent with prior research suggesting a potential link between neural connectivity and behavioral measures, which may be more significant or severe in clinical samples (e.g., Zhang et al., 2023). However, our findings are consistent with prior work on the neuronal effects of mindfulness training and extend the existing literature by suggesting that these relationships may not always be evident, at least not in an intervention study of typically developing adolescents without mental health diagnoses (e.g., due to possible floor effects, reliance on self-report measures, or a need for longer and more frequent intervention). It is also possible that behavioral effects of NAMT may take longer to develop than the neural effects, and the timing of such changes could be examined in future longitudinal studies. Further, these analyses could have been impacted by restriction of range (i.e., floor effects) and/or limited power. Thus, while our results appear promising, more research is needed to demonstrate that the observed connectivity changes are associated with beneficial behavioral and cognitive outcomes, clarify the duration of NAMT effects, and explore the applicability to clinical populations.

### 4.1 Limitations and future directions

Despite its novelty and strengths, this pilot study is not without limitations. First, the absence of a randomized sham or control condition limits our ability to distinguish the causal impact of the NAMT task from placebo effects or other state-related changes (e.g., participant stress or fatigue). Furthermore, five participants were excluded from analyses due to high rates of head motion during rs-fMRI, resulting in a smaller sample size and possibly reduced generalizability of results as it has been demonstrated that youth with lower average motion during rs-fMRI may differ in sociodemographic characteristics from those with higher motion (e.g., by being older and having higher socioeconomic status; Cosgrove et al., 2022). Relatedly, the sample consisted of typically developing adolescents without mental illness, so the applicability of the findings to individuals with psychiatric diagnoses and/or developmental differences remains uncertain. Future research should therefore aim to replicate the present findings in larger and more diverse samples, including clinical populations, using a control group. Lastly, the study assessed functional connectivity of the PCC immediately before and after one session of the NAMT task and did not evaluate more global changes in connectivity across the whole brain or long-term changes in connectivity. It is therefore unclear whether the observed results are sustained over time and whether dose-dependent effects may be observed with more sessions of NAMT. To address this, future work should include longitudinal studies of NAMT that (1) investigate the persistence of changes in functional connectivity over time and (2) examine whether multiple NAMT sessions have a greater impact on connectivity. Exploring the relationship between changes in connectivity and behavioral outcomes, such as improvements in emotion regulation or mindfulness skills, would also enhance our understanding of mechanisms of action for mindfulness training.

## 5 Conclusion

The present investigation contributes to the newly growing body of literature on mindfulness training with neurofeedback by demonstrating that this approach may influence resting-state functional connectivity in typically developing adolescents. Specifically, completion of our NAMT task was associated with increased connectivity between the PCC seed and regions involved in emotional processing and memory. These findings provide preliminary evidence of the potential benefits of the NAMT task for promoting more self-awareness of emotions and experiences. However, further research is needed to expand upon these findings and fully elucidate the mechanisms underlying the effects of the NAMT task on functional connectivity and mental health outcomes.

## Data Availability

The raw data supporting the conclusions of this article will be made available by the authors, without undue reservation.

## Footnotes

^1^https://picsl.upenn.edu/software/ants/
